# Comprehensive Evaluation of Corticospinal Tract Metabolites in Amyotrophic Lateral Sclerosis Using Whole-Brain ^1^H MR Spectroscopy

**DOI:** 10.1371/journal.pone.0035607

**Published:** 2012-04-23

**Authors:** Varan Govind, Khema R. Sharma, Andrew A. Maudsley, Kristopher L. Arheart, Gaurav Saigal, Sulaiman Sheriff

**Affiliations:** 1 Department of Radiology, University of Miami School of Medicine, Miami, Florida, United States of America; 2 Department of Neurology, University of Miami School of Medicine, Miami, Florida, United States of America; 3 Department of Epidemiology, University of Miami School of Medicine, Miami, Florida, United States of America; University of Cambridge, United Kingdom

## Abstract

Changes in the distribution of the proton magnetic resonance spectroscopy (MRS) observed metabolites N-acetyl aspartate (NAA), total-choline (Cho), and total-creatine (Cre) in the entire intracranial corticospinal tract (CST) including the primary motor cortex were evaluated in patients with amyotrophic lateral sclerosis (ALS). The study included 38 sporadic definite-ALS subjects and 70 age-matched control subjects. All received whole-brain MR imaging and spectroscopic imaging scans at 3T and clinical neurological assessments including percentage maximum forced vital capacity (FVC) and upper motor neuron (UMN) function. Differences in each individual metabolite and its ratio distributions were evaluated in the entire intracranial CST and in five segments along the length of the CST (at the levels of precentral gyrus (PCG), centrum semiovale (CS), corona radiata (CR), posterior limb of internal capsule (PLIC) and cerebral peduncle (CP)). Major findings included significantly decreased NAA and increased Cho and Cho/NAA in the entire intracranial CST, with the largest differences for Cho/NAA in all the groups. Significant correlations between Cho/NAA in the entire intracranial CST and the right finger tap rate were noted. Of the ten bilateral CST segments, significantly decreased NAA in 4 segments, increased Cho in 5 segments and increased Cho/NAA in all the segments were found. Significant left versus right CST asymmetries were found only in ALS for Cho/NAA in the CS. Among the significant correlations found between Cho/NAA and the clinical assessments included the left-PCG versus FVC and right finger tap rate, left -CR versus FVC and right finger tap rate, and left PLIC versus FVC and right foot tap rate. These results demonstrate that a significant and bilaterally asymmetric alteration of metabolites occurs along the length of the entire intracranial CST in ALS, and the MRS metrics in the segments correlate with measures of disease severity and UMN function.

## Introduction

Amyotrophic lateral sclerosis (ALS) is a neurodegenerative disease characterized by progressive degeneration of both upper motor neurons (UMN) in the primary motor cortex (PMC) and lower motor neurons (LMN) in the brain stem and spinal cord anterior horns. Despite identification of pathologies in the PMC and corticospinal tracts (CST) of autopsy tissue samples with ALS [1], [2], there are no biomarkers identified to date that reliably indicate presence of such pathologies in the brain of patients with ALS. In an effort to find neuroimaging biomarkers indicative of UMN degeneration, advanced MR techniques (e.g., magnetic resonance spectroscopy, diffusion tensor imaging and functional MRI) have been explored [3], [4]. It is hypothesized that the neuropathologically proven motor neuron and CST degeneration in ALS might occur gradually with no apparent manifestation of macroscopic tissue structural changes in the early stage that are detectable by conventional MRI methods. In contrast, neurochemicals indicative of metabolic processes responsible for degeneration of the motor neurons and CST in patients with ALS can be accessed from the disease onset stage using proton MR spectroscopic (MRS) methods. Proton MRS enables non-invasive measurement of brain metabolites [5] such as N-acetyl aspartate (NAA; a marker of viability of neurons), total-choline (Cho; an indicator of cell membrane structural integrity, synthesis and degradation; MRS signal sum of all choline-moiety containing compounds), and total-creatine (Cre; an indicator of cellular energetics; MRS signal sum of creatine and phosphocreatine). Several previous cross-sectional and longitudinal studies have demonstrated the value of proton MRS for evaluation of metabolite alterations in the PMC [6], [7], [8], [9], [10], [11], [12], [13], [14], [15], [16] and CST [11], [17] of patients with ALS using single voxel (SVS) and 2D MR spectroscopic imaging [11], [17], [18], [19] (MRSI) methods. In general, most of these studies have reported significantly altered metabolite concentrations or ratios in the motor pathways of patients with ALS.

The data acquisition in these studies has been based on manual selection of relevant anatomical regions such as PMC and intracranial CST in the case of SVS and spectra from voxels within manually selected ROIs in the case of MRSI. Manual selection of anatomical regions in longitudinal studies is susceptible to user-subjectivity, increasing the probability of error in obtaining data from the same anatomical volume in follow up studies. This is then likely to contribute to increased variance in group comparisons and follow-up studies.

The objective of this methodological study was to develop user-independent and comprehensive MRSI data acquisition and analysis methods for evaluating metabolite changes in the intracranial CST in patients with ALS. Towards achieving this, our unique volumetric proton MRSI acquisition method was used to acquire data from the whole-brain of patients with ALS and controls. A new atlas-based data analysis approach was introduced to evaluate metabolite values in the entire 3D-anatomical volume of the intracranial CST (the length of the tract from the PMC to the cerebral peduncle). Furthermore, the length of the CST was segmented into five discrete but contiguous anatomical regions to evaluate metabolite variations in each of the segments and to assess if any anatomical segment has degraded preferentially over the others in ALS. Finally, metrics from the metabolite and clinical measures were correlated to assess associations between the measures. Our specific hypothesis is that metabolite analysis in patients with ALS using the entire volume of the intracranial CST and its segments will provide new insight on the extent of metabolite changes along the intracranial CST bilaterally and the association between metabolite measures in the CST (intracranial CST as a whole and its segments) and clinical measures.

## Methods

### Subject Selection

Thirty eight subjects (52.3±8.8 years; 26 males; 4 left handed) that met the revised El Escorial criteria [20] for sporadic definite-ALS were recruited from the Kessenich Family MDA ALS Center, University of Miami for inclusion in this study. Though all consecutive subjects with ALS were screened for participation in this study, only those who met the standard 3T MRI pre-screening procedure for scanning and were healthy enough to lie on their back with minimal body motion for the duration of the MRI scan were selected for this study. Sixteen subjects were treated with riluzole either before participating in this study or at the time of scan, and the duration of their riluzole treatment varied between 2 weeks and 32 months prior to the scan. For comparison, data from a group of 70 control subjects (51.9±6.3 years; 27 males) were included and all were recruited from the local community. For selecting control subjects, standard MRI prescreening procedures were used to exclude subjects with MRI contraindications, previous brain injury or surgery, or a history of neurological disease, psychiatric illness or substance abuse. The study protocol was approved by the institutional review board of University of Miami, and all subjects provided signed written consent before participation.

### Neurological Assessments

Subjects with ALS received detailed physical and neurological examinations by a neurologist (KRS). The clinical assessment included evaluating hand and leg muscle stretch reflexes, muscle tone, disease severity and upper motor neuron function. Disease severity was evaluated using the ALS Functional Rating Scale-Revised (ALSFRS-R; [21]) and percentage of predicted forced vital capacity (% FVC). The ALSFRS-R score ranges from 0 to 48 with 0 and 48 indicating total disability and no disability, respectively. The functional status of the pyramidal tracts, an indicator of upper motor neuron disability, was quantified by counting the number of rapid foot taps, finger taps, lip movements with *‘pa-pa’* syllable repeats and tongue movements with *‘la-la’* syllable repeats, each in 10 s [22], [23], and the counts were converted to rates (per second scores). The above measurements were performed twice during the same session with an interval of 1 minute in between for obtaining the representative average rate for each of the muscle sites assessed.

For the remainder of this paper, measures of UMN function constitute the rates of rapid foot and finger taps, lip movements with *‘pa-pa’* syllable repeats and tongue movements with ‘*la-la’* syllable repeats, measures of disease severity include the ALSFRS-R score and % maximum of predicted FVC, clinical measures indicate measures of UMN function and disease severity, and MRS metrics include concentrations of NAA, Cre and Cho (provided in institutional units) and Cho/NAA ratio.

### Magnetic resonance imaging and spectroscopy

MR data were acquired at 3 Tesla (Siemens TimTrio) using an eight-channel phased-array head coil for detection. An hour-long MR protocol was used, consisting of structural MRI (T1-weighted), diagnostically relevant MRI and whole-brain MRSI acquisitions. The MRI sequences included T1-weighted (MPRAGE; TE/TR: 2.38 ms/2300 ms, TI: 900 ms, 160 slices, 1 mm^3^ isotropic resolution, and ∼5.2 min.), FLAIR (TE/TR: 101 ms/9 s, 33 slices, 4 mm slice thickness, field of view (FOV): 220×220 mm^2^, and ∼2.2 min.) and T2-weighted turbo spin-echo MRIs (TE/TR: 87 ms/6.4 s, 33 slices, 4 mm slice thickness, FOV 220×220 mm^2^, and ∼2.2 min.).

MRSI data were acquired using a volumetric spin-echo EPSI sequence (TR/TE: 1710 ms/70 ms, FOV: 280×280×180 mm^3^, 100×50×18 spatial points, 135 mm excitation slab, and acquisition time of 26 min.). The sequence acquires two datasets, a spin-echo metabolite dataset and a low flip-angle (20°) gradient-echo water reference dataset. It includes frequency-selective water suppression, and inversion recovery nulling of the extra-cranial lipid signal with a TI of 198 ms. Additional details of the data acquisition were provided in our earlier publications [23], [24].

### Data processing and analysis

MRSI data were processed using the MIDAS package [25]. Briefly, data imported into the MIDAS environment went through a pipeline of processing steps that included formation of volumetric metabolite images, co-registration of T1-MRI and MRSI, segmentation of T1-MRI, calculation of MRSI voxel tissue content based on segmentation of the T1-weighted MRI, signal normalization to institutional units using the tissue water reference signal and tissue content information from the same voxel, and spatial registration to the Montreal Neurological Institute (MNI) single-subject MRI template [26] at 2 mm^3^ isotropic resolution. For spectral fitting, metabolite prior information for NAA, Cre and Cho were simulated using an in-house developed program that used the GAMMA library [27] and published chemical shifts and coupling constants [5]. The nominal MRSI voxel volume as acquired was approximately 0.3 mL (5.6×5.6×10 = 314 mm^3^). After zero-padding the data to a 64×64×32 matrix and applying Gaussian spatial smoothing in the three orthogonal directions, the voxel volume used for data analysis was approximately 1 mL.

Metabolite data analysis was performed in the MNI template space using a single-subject probabilistic CST atlas [28], [29]. From this atlas, only voxels with the probability values >0.20 were included to create a modified atlas for data analysis. Furthermore, it was divided into five segments at anatomically appropriate levels in the superior-inferior direction, namely, precentral gyrus (PCG), centrum semiovale (CS), corona radiata (CR), posterior limb of internal capsule (PLIC) and cerebral peduncle (CP). Metabolite values (NAA, Cre, Cho) and Cho/NAA ratio in voxels within the right and left side of the entire intracranial CST atlas and its five segments were obtained. For each of the regions in the atlas, metabolite values were first corrected for partial volume signal loss due to CSF contribution at each voxel and then, mean metabolite values and ratios were calculated.

For statistical analyses, three data selection criteria were applied to ensure the quality of the data chosen. First, a line-width criterion was applied to only include voxels with fitted spectral line-width between 2 and 12 Hz. Next, a fractional tissue volume criterion was applied to only include voxels with underlying tissue volume of ≥70%. Finally, an outlier exclusion criterion was applied to exclude voxels with metabolite values greater than three times the standard deviation of the corresponding metabolite value over all the voxels within the particular region. This last criterion was applied separately for each metabolite value and ratio.

### Statistical tests

The metabolite measures (NAA, Cho, Cre, and Cho/NAA) of the ALS and control subject groups in both the entire intracranial CST and its segments in each hemisphere were compared using analysis of covariance (ANCOVA) test. To control for possible confounding effects of age and gender, we included effects for age, gender, and the interactions of these two variables with group. After testing the full model, we found no significant interactions of group and gender for any of the analyses. Therefore, this interaction was deleted from subsequent models. Significant interactions were found for group and age in several analyses; therefore we included separate effects for age dependent on group, i.e. we nested age in group to provide a separate slopes model.

We used a linear mixed model to perform a repeated measures ANOVA to evaluate left intracranial-CST versus right intracranial-CST metabolite asymmetries separately for the entire intracranial CST and its segments. The same test was used to evaluate differences in metabolite values between the segments along the intracranial CST in each hemisphere. The model included group (ALS vs. Control), age nested in group (separate slopes model), and gender as fixed effects and person nested in group as the random effect.

For evaluating associations between Cho/NAA and the measures of UMN function and disease severity, Pearson's product moment partial correlation was used, and the results are reported as correlation coefficients (r-values) between the scores or values of the two measures. Handedness information was not included in this test as the patient population was overwhelmingly right-handed (34 right-handed and 4 left-handed), making any results based on handedness unstable.

P-values of <0.05 were considered to be significant for all the tests performed. To account for multiple testing, an appropriate Bonferroni correction was applied to the p-values of the tests performed to adjust for the four metabolite data measures (NAA, Cre, Cho and Cho/NAA) in each of the regions, two hemispheric CST (left, right) and five anatomical segments (PCG, CS, CR, PLIC and CP) within each hemispheric CST (i.e., px4 for the between group CST metabolite comparisons in each hemisphere, or px4x2 for the left-CST versus right-CST metabolite comparisons in each group, and px4x5 for the comparisons between the segments in each hemispheric CST in each group), thereby maintaining the same significance level (p<0.05).

## Results

### Disease duration, severity and metrics of clinical measures

Disease duration and severity (ALSFRS-R and FVC) of the patients in the ALS group are provided in Table 1. Disease durations were calculated from the symptom onset date to the date of the MRI scan. The clinical measures of motor neuron function for the patients in the ALS group are provided in Table 2.

**Table 1:** Disease duration and severity of the patients in the ALS group.

Measure	Description	Value Type	ALS Group (n = 38)
Disease duration (months)	since noticing symptoms	mean±SD	29±27
		median	18
		range	2–120
	since diagnosis	mean±SD	18±23
		median	7
		range	1–83
ALSFRS-R*	score	mean±SD	37±7
		median	37
		range	22–48
Forced vital capacity	% predicted	mean±SD	79±18
		median	80
		range	41–125

* Amyotrophic lateral sclerosis functional rating scale-revised.

**Table 2:** Clinical measures of motor neuron function in the ALS group.

Measure	Description	Value Type	ALS Group (n = 38)
Handedness	right handed	number of subjects	34
	left handed	number of subjects	4
Finger tapping rate*	right hand	mean±SD	2.5±0.9
		median	2.5
		range	0.9–4.2
	left hand	mean±SD	2.5±0.9
		median	2.5
		range	0.7–4.1
	mean of right and left	mean±SD	2.5±0.7
		median	2.5
		range	0.9–4.1
Foot tapping rate*	right leg	mean±SD	2.1±1.1
		median	2.2
		range	0.0–4.7
	left leg	mean±SD	2.0±1.1
		median	2.0
		range	0.0–5.1
	mean of right and left	mean±SD	2.1±1.1
		median	2.1
		range	0.0–4.9
*La-la* syllable repeat rate*	tongue muscle	mean±SD	3.4±1.1
		median	3.4
		range	0.8–5.0
*Pa-pa* syllable repeat rate*	lip muscle	mean±SD	3.4±0.9
		median	3.4
		range	0.9–5.1
Bulbar muscle syllable repeat rate*	tongue and lip muscles**	mean±SD	3.4±1.0
		median	3.5
		range	0.8–5.0

* Rates are provided in s^−1^;

** mean of *la-la* and *pa-pa* syllable repeat rates.

### Spectral quality and CST atlas

**Figure 1:**
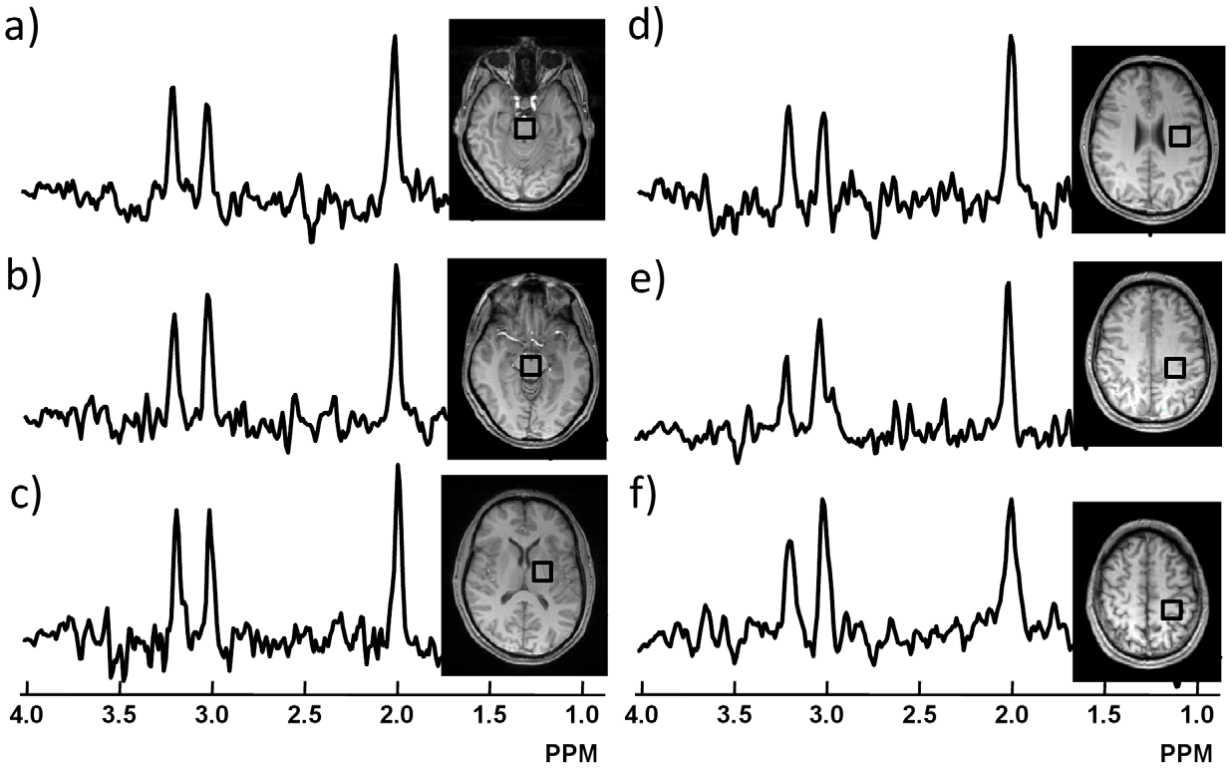
Representative ^1^H-MR spectra in the brain of a patient with ALS. ^1^H-MR spectra obtained at 3T from six anatomical regions (square boxes on the MRIs) along the length of the corticospinal tract in the brain of a patient with ALS (male, 43 years) are shown. (a: medulla; b: pons/midbrain, c: left internal capsule; d: left corona radiata; e: left centrum semiovale; f: left precentral gyrus/subcortical white matter). The spectra shown were obtained by averaging spectra from 2.7 mL to 6.0 mL contiguous voxels in that region.

Representative 1H MR spectra obtained from six anatomical regions along the length of the CST between the precentral gyrus and cerebral peduncle of a patient with ALS (male, 43 year old) are shown in Figure 1. Each spectrum was obtained by averaging over a number of contiguous voxels in the same region. As seen in the figure, the quality of spectra is good across all regions, including from the medulla and pons/midbrain.

**Figure 2:**
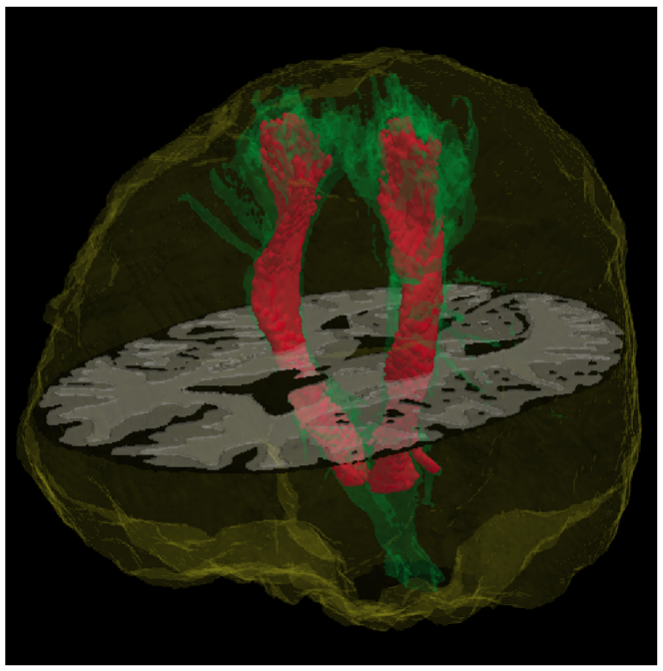
Intracranial corticospinal tract atlas. The full-range probabilistic atlas is shown in green. We modified this atlas (shown in red) for our use by including only voxels with probability values greater than 0.20 and by excluding voxels inferior to the cerebral peduncle. The axial MRI image is included to provide anatomical context.

In Figure 2 are shown the entire intracranial CST atlas [28], [29] and its modified version used for data analysis. The probability cutoff used (>0.20) is seen to limit the neuronal WM fiber arborization, thereby including only the fiber bundles that are definitively part of the CST for analysis.

### Metabolite differences between the ALS and control groups in the CST

**Figure 3:**
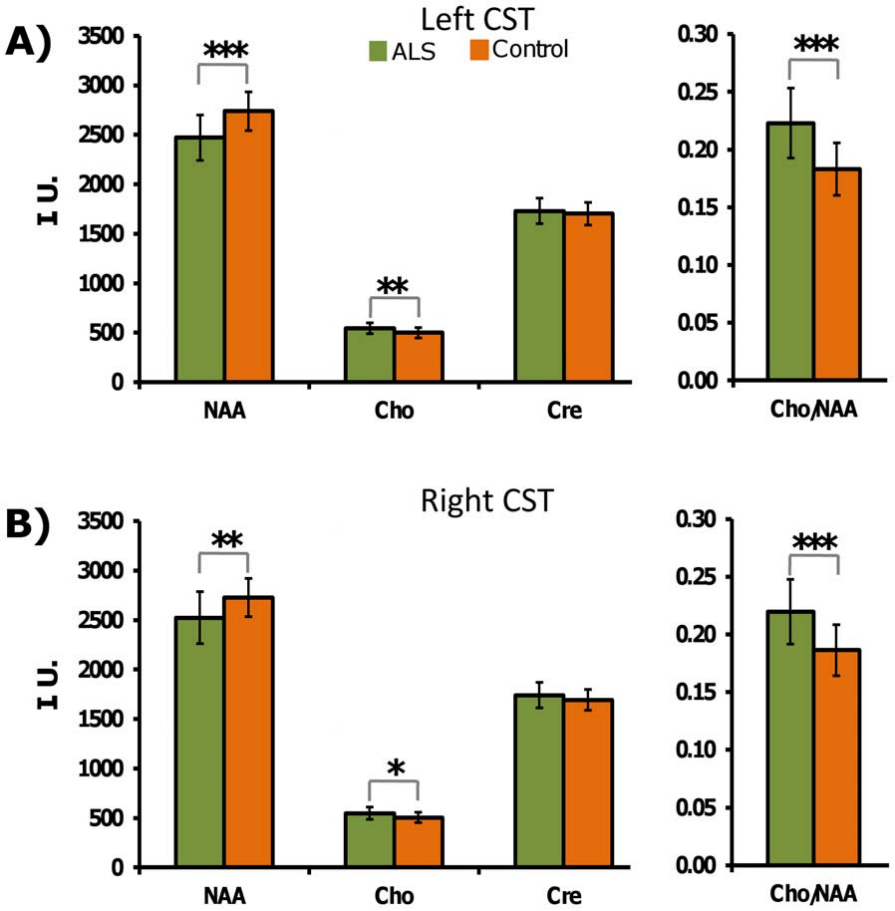
Comparison of intracranial corticospinal tract (CST) metabolite values between the ALS and control groups. Comparisons of the left (A) and right (B) entire intracranial corticospinal tract (CST) metabolite values (mean ± SD) in the ALS (n = 38) and control (n = 70) groups (age range: 34–65 years) using ANCOVA. The green and orange histograms indicate the metabolite values of the entire intracranial CST in the ALS and control groups, respectively. The asterisks indicate significant group differences with *p<0.05, **p<0.005 and ***p<0.0001. The significant percent differences found were: −8.9 (left) and −6.2 (right) in NAA, +8.1 (left) and +7.5 (right) in Cho, and +20.0 (left) and +15.7 (right) in Cho/NAA. The abbreviations used indicate as NAA: N-acetyl aspartate; Cre: total-creatine; Cho: total-choline; I. U.: institutional units.

In Figure 3 are shown comparisons of the metabolite values and Cho/NAA ratio for the ALS and control groups for the entire intracranial CST. As shown, significant group differences (indicated by asterisks) were observed for NAA, Cho and Cho/NAA in both the left and right entire intracranial CST whereas there were no significant differences for Cre. The significant percent metabolite differences found in the ALS group as compared to the control subject group were: −8.9% (left) and −6.2% (right) for NAA, 8.1% (left) and 7.5% (right) for Cho, and 20.0% (left)and 15.7% (right) for Cho/NAA, respectively.

**Figure 4:**
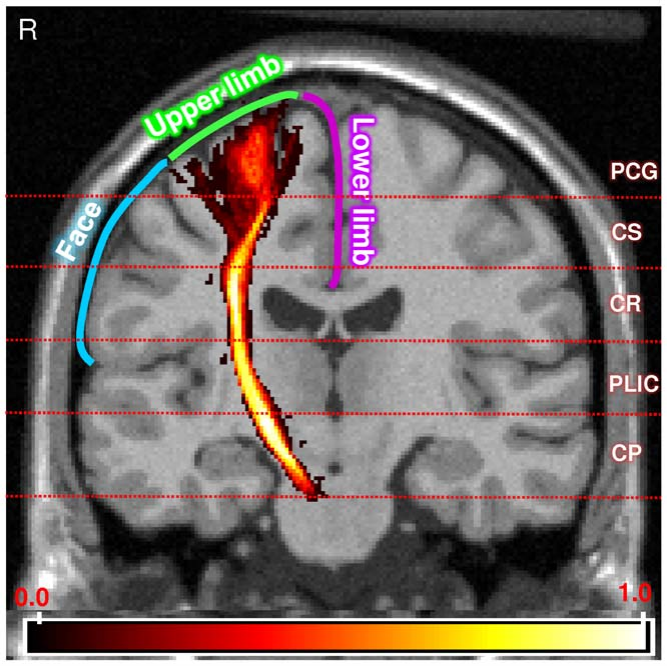
Segmented intracranial corticospinal tract (CST) atlas and the somatotopic arrangement within the primary motor cortex. The probabilistic corticospinal tract (CST) atlas is overlaid onto the coronal orientation T1-MRI template (Montreal Neurological Institute). The color bar indicates the full range of the probability values in the CST atlas. The horizontal lines indicate the approximate anatomical levels at which the five segments were made along the length of the CST. PCG: Precentral gyrus, CS: Centrum semiovale, CR: Corona radiata, PLIC: Posterior limb of the internal capsule, CP: Cerebral peduncle and R: Right side of the subject. The blue, green and purple lines indicate the somatotopic arrangement within the primary motor cortex that are associated with the face and lower and upper limb parts, respectively.

**Figure 5:**
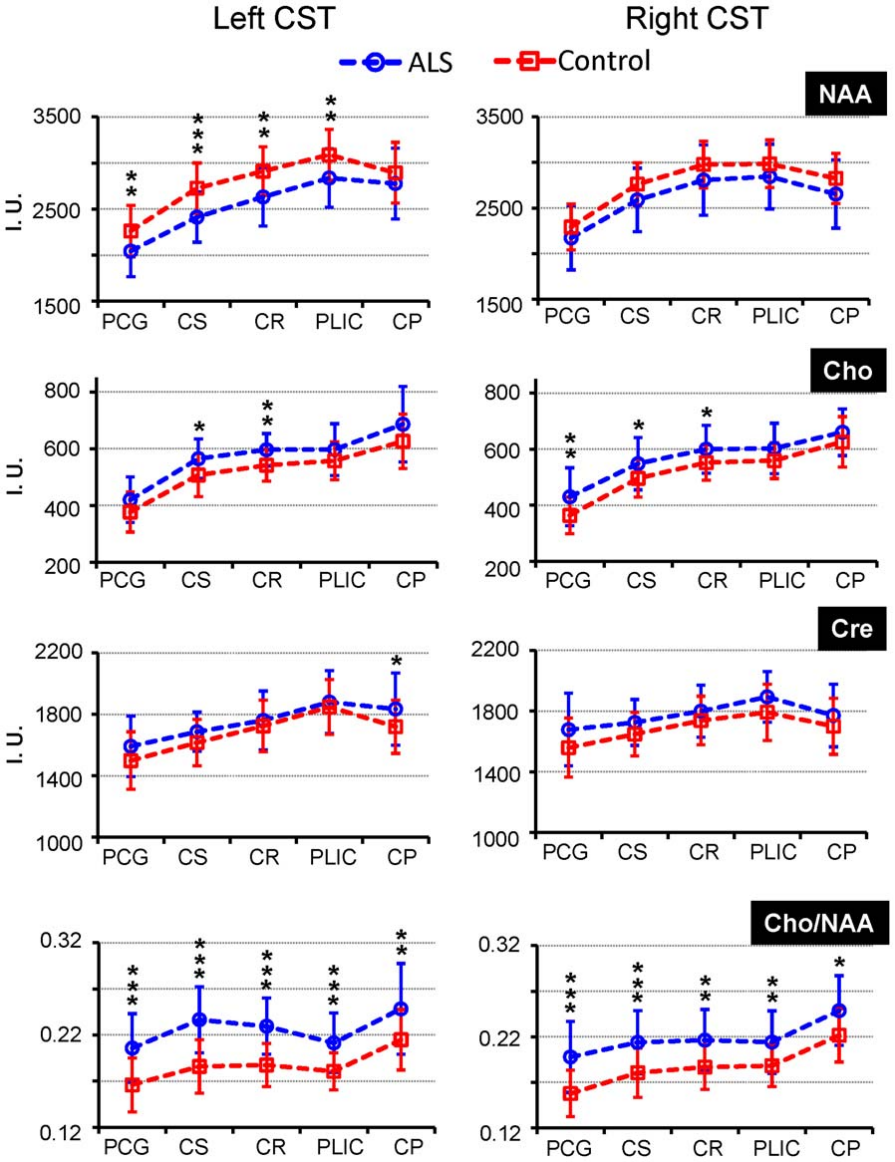
Metabolite metrics in the length of the intracranial corticospinal tracts in the ALS and control groups. Metabolite metrics along the length of the intracranial corticospinal tracts (CST) in patients with ALS (blue; n = 38) and control (red; n = 70) groups in the age range of 34–65 years using ANCOVA. The asterisks indicate significant group differences with *p<0.05, **p<0.005 and ***p<0.0001. The abbreviations used indicate as NAA: N-acetyl aspartate, Cho: total-choline, Cre: total-creatine, PCG: precentral gyrus, CS: centrum semiovale, CR: corona radiata, PLIC: posterior limb of internal capsule, CP: cerebral peduncle, and I.U.: institutional units.

In Figure 4 are shown the five contiguous segments (PCG, CS, CR, PLIC and CP) along the length of the CST between the PMC and cerebral peduncle, and a simplified motor homunculus indicating the somatotopic arrangement for the face and upper and lower limbs. In Figure 5 are shown comparisons of the metabolite metrics obtained in the five segments of the bilateral intracranial CST of the ALS and control groups. In general, there was a consistent bilateral trend of either increased (Cho or Cho/NAA) or decreased (NAA) metabolite values in the ALS group as compared to the control group. Significant group differences were noted for NAA in 4 of the 5 segments in the left CST (except in the CP), for Cho in 2 segments in the left CST (in the CS and CR) and 3 segments in the right CST (in the PCG, CS and CR), and for Cho/NAA in all the segments of CST bilaterally. The significant percentage differences observed for Cho/NAA ranged from +9.5% in the CP of the right CST to +24.5% in the CS of the left CST.

### Inter and intra-hemispheric differences for metabolites in the intracranial CST

#### Comparisons between the left and right hemisphere

There were no significant metabolite differences in the entire intracranial CST (data not shown) in both the ALS and control subject groups. However, in the segments of the intracranial CST (data not shown), there was a significant left versus right difference for Cho/NAA in the CS segment in the ALS group (p = 0.036), and no significant metabolite differences in any of the segments in the control group.

#### Comparisons between the segments within each hemisphere

In Tables 3 and 4 are shown the p-values of the between segments metabolite comparisons in each hemispheric CST for the control and ALS subject groups, respectively (mean±SD are shown in Figure 5). Significant metabolite differences were noted for 34 out of 40 possible comparisons in the left (p = 0.031 to <0.0001) and 30 in the right hemispheric segments (p = 0.015 to <0.0001) for the controls, and 24 in the left (p = 0.043 to <0.0001) and 17 (p = 0.030 to <0.0001) in the right hemispheric segments for the ALS group.

**Table 3:** P-values of the metabolite comparisons between the intracranial CST segments in each hemisphere in the control group.

Metabolite	Region	Control Left Intracranial-CST				Control Right Intracranial-CST			
		CP	CR	CS	PCG	CP	CR	CS	PCG
**NAA**	**CP**								
	**CR**	1				0.015*			
	**CS**	0.016*	0.001*			1	<0.0001*		
	**PCG**	<0.0001*	<0.0001*	<0.0001*		<0.0001*	<0.0001*	<0.0001*	
	**PLIC**	0.003*	0.001*	<0.0001*	<0.0001*	0.010*	1	<0.0001*	<0.0001*
**Cho**	**CP**								
	**CR**	<0.0001*				<0.0001*			
	**CS**	<0.0001*	0.031*			<0.0001*	<0.0001*		
	**PCG**	<0.0001*	<0.0001*	<0.0001*		<0.0001*	<0.0001*	<0.0001*	
	**PLIC**	<0.0001*	1	<0.0005*	<0.0001*	<0.0001*	1	<0.0001*	<0.0001*
**Cre**	**CP**								
	**CR**	1				1			
	**CS**	0.007*	0.001*			1	0.007*		
	**PCG**	<0.0001*	<0.0001*	0.001*		0.001*	<0.0001*	0.030*	
	**PLIC**	0.001*	<0.0005*	<0.0001*	<0.0001*	0.075	1	<0.0001*	<0.0001*
**Cho/NAA**	**CP**								
	**CR**	<0.0001*				<0.0001*			
	**CS**	<0.0001*	1			<0.0001*	1		
	**PCG**	<0.0001*	<0.0001*	0.001*		<0.0001*	<0.0001*	<0.0001*	
	**PLIC**	<0.0001*	0.986	1	0.007*	<0.0001*	1	1	<0.0001*

Significant p-values (<0.05) are indicated by asterisks (*). Out of a total of 40 possible comparisons, 34 and 30 between-segment comparisons were found to be significant in the left and right CST, respectively. The abbreviations used are: CST: corticospinal tract, PCG: precentral gyrus, CS: centrum semiovale, CR: corona radiate, PLIC: posterior limb of internal capsule, and CP: cerebral peduncle, NAA: N-acetyl aspartate, Cho: total-choline, Cre: total-creatine.

**Table 4:** P-values of the metabolite comparisons between the intracranial CST segments in each hemisphere in the ALS group.

Metabolite	Region	ALS Left Intracranial-CST				ALS Right Intracranial-CST			
		CP	CR	CS	PCG	CP	CR	CS	PCG
**NAA**	**CP**								
	**CR**	1				1			
	**CS**	<0.0001*	0.027*			1	0.170		
	**PCG**	<0.0001*	<0.0001*	<0.0001*		<0.0001*	<0.0001*	<0.0001*	
	**PLIC**	1	0.089	<0.0001*	<0.0001*	0.400	1	0.026*	<0.0001*
**Cho**	**CP**								
	**CR**	0.003*				0.030*			
	**CS**	<0.0001*	0.557			<0.0001*	0.209		
	**PCG**	<0.0001*	<0.0001*	<0.0001*		<0.0001*	<0.0001*	<0.0001*	
	**PLIC**	0.014*	1	1	<0.0001*	0.063	1	0.168	<0.0001*
**Cre**	**CP**								
	**CR**	1				1			
	**CS**	0.015*	0.907			1	0.838		
	**PCG**	<0.0001*	0.003*	0.234		1	0.200	1	
	**PLIC**	1	0.155	<0.0001*	<0.0001*	0.080	0.272	<0.0001*	<0.0001*
**Cho/NAA**	**CP**								
	**CR**	0.928				0.001*			
	**CS**	1	1			<0.0005*	1		
	**PCG**	<0.0005*	0.043*	0.004*		<0.0001*	0.492	1	
	**PLIC**	0.002*	0.226	0.022*	1	<0.0005*	1	1	0.970

Significant p-values (<0.05) are indicated by asterisks (*). Out of a total of 40 possible comparisons, 24 and 17 between-segment comparisons were found to be significant in the left and right CST, respectively. The abbreviations used are: CST: corticospinal tract, PCG: precentral gyrus, CS: centrum semiovale, CR: corona radiate, PLIC: posterior limb of internal capsule, and CP: cerebral peduncle, NAA: N-acetyl aspartate, Cho: total-choline, Cre: total-creatine.

### Associations between the metabolite and clinical measures

In the entire intracranial CST of the ALS group, significant negative correlations were observed between Cho/NAA in the left CST versus FVC (r = −0.332, p = 0.042), and Cho/NAA in the bilateral CST versus right finger tap rate (left side: r = −0.589 6, p = <0.0001; right side: r = −0.490 5, p = 0.002). The correlation between Cho/NAA in the right CST and left finger tap rate also approached towards significance (r = −0.304, p = 0.064).

In the segments of the CST of the ALS group, the significant correlations noted between Cho/NAA and the measures of UMN function and disease severity were: the left PCG versus FVC (r = −0.412, p = 0.010) and right finger tap rate (r = −0.502, p = 0.001), the left CS versus FVC (r = −0.367, p = 0.023) and right finger tap rate (r = −0.485, p = 0.002), and the left PLIC versus FVC (r = −0.327, p = 0.045) and right foot tap rate (r = −0.326, p = 0.046).

There were no other significant correlations between Cho/NAA in the entire intracranial CST and its segments and other clinical measures such as foot tap rates, syllable repeat rates, etc.

## Discussion

In this study a comprehensive evaluation of ^1^H-MRS observed cerebral metabolite alterations in the primary motor cortex and corticospinal tract of patients with ALS was performed. For the purpose of finer analysis of metabolite alterations, the intracranial CST was divided into 5 anatomically distinct segments. Data analysis was performed at the volume levels of the entire intracranial CST and its segments bilaterally. Furthermore, correlations between the MRS measures and the clinical measures of UMN function and disease severity were evaluated to find associations between the measures.

The main findings of this study were: (1) Data from the whole anatomical volume of the primary motor cortex and CST in controls and patients with ALS can be obtained conveniently using a whole-brain MRSI acquisition method; the availability of whole-brain MRSI data enabled us to use an intracranial CST atlas that eliminated user-subjectivity in selecting volumes (or ROIs) for metabolite evaluation. (2) Significant changes in NAA, Cho, Cho/NAA of the entire intracranial CST in the ALS group, and on a finer level, significant NAA decreases in 4 of the 5 segments in the left CST, significant Cho increases in 2 and 3 segments in the left and right intracranial CST, respectively, and significant Cho/NAA increases in all the CST segments bilaterally. (3) A significant left versus right intracranial CST difference for Cho/NAA in the CS segment in the ALS group. (4) Fewer number of comparisons of metabolites between the segments in each hemispheric CST showed significant differences in ALS as compared to controls. (5) Significant correlations between Cho/NAA in the entire intracranial CST of patients with ALS and their disease severity or UMN function measures (FVC and finger tap rate), and on a finer level, significant correlations between Cho/NAA of 3 intracranial CST segments (left-PCG, left-CS and left-PLIC) and disease severity and UMN function measures.

Previous single voxel [6], [9], [13], [30] and MRSI [11], [16], [18], [19] studies in patients with ALS have manually selected the anatomical locations for data acquisition and/or analysis along the length of CST between the primary motor cortex and the cerebral peduncle. Such a data acquisition or analysis approach has inherent user-subjectivity and inevitably includes data from non-motor pathways, both of which are likely to contribute to increased variance in group comparison studies. In contrast, the volumetric MRSI acquisition method used in this study acquired data from the whole-brain encompassing the entire bilateral intracranial CST. Furthermore, an intracranial CST atlas was used for data analysis, which eliminated user-subjectivity in selection of the volumes for evaluation. This comprehensive and template based approach is well suited for evaluating the spatial and temporal changes in metabolites in longitudinal and treatment efficacy studies in ALS and other brain pathologies.

The results from this study cannot directly be compared with previous studies in literature as no studies have used the approach of whole-brain data acquisition and template based analysis, apart from the fact that the acquisition sequence parameters used in this study (specifically, the echo time) are different from those used in previous studies. However, in general terms, our results agree with most previous MRS studies that have evaluated the cerebral metabolite concentration or ratio changes, at least in a part of the intracranial CST in patients with ALS. In the case of NAA and NAA/Cho, a few previous studies [9], [10], [12], [14], [15], [16], [18], [19], [31], [32], [33] have reported results similar to ours. In our data analysis, significant changes in NAA or Cho/NAA were found in the entire intracranial CST of patients with ALS (Figure 3). The percentage differences of metabolite values in ALS as compared to controls ranged from −6.2% to −8.9% for NAA and +15.7% to +20.0% for Cho/NAA. In an intra-subject MRSI reproducibility study [34] that used the same acquisition sequence as this study, the coefficients of variation (COVs) reported were 1.3% and 1.9% for NAA in the frontal and parietal lobes, respectively, and 2.9% and 2.7% for Cho/NAA in the same two lobar regions, respectively. At the spatial resolution of the whole-brain MRSI data used for analysis in this study (∼1 mL), the intracranial CST can be considered to be co-localized in both frontal and parietal lobes of the brain. Thus, it can be inferred from the above COV information that the percentage metabolite differences observed between the ALS and control groups in this study would remain significant even after accounting for the test-retest variability of the MRS metrics used in this study.

NAA is present almost exclusively in neurons [5], [35], and so the decrease in its concentration in a brain region is indicative of reduced neuronal density due to its loss or reduced neuronal cell volume due to progressive neurodegeneration or metabolically dysfunctional albeit structurally intact neurons. The neuropathological features found in the primary motor cortex and CST of most ALS autopsy cases include loss of motor neurons, intracytoplasmic inclusions and cytoplasmic shrinkage in surviving motor neurons, atrophy of the motor cortex, and degeneration of the CST [1], [2]. The reduced NAA found in patients with ALS in this study is consistent with the above mentioned autopsy findings in ALS.

Significantly increased Cho was found bilaterally in the entire intracranial CST of patients with ALS (Figure 3). The increased Cho ranged from +7.5% to +8.1%. Similar to this study, previous MRS studies in the motor cortex (or the precentral gyrus) of patients with ALS have also reported significantly increased Cho [6] or Cho/Cre [10], [14], [19], [33], [36].

The proton MR observed Cho signal in the brain has major contributions from the membrane constituents, glycerophosphocholine and phosphocholine, and a minor contribution from free choline [5]. In ALS, neuropathological studies [1], [2] have found loss of large myelinated white matter fibers together with variable astrocytic gliosis and lipid laden macrophages in the degenerated primary motor tracts. Our results suggest that patients in the early stage of the disease are likely to have ongoing inflammation in their motor pathways; presence of such an inflammatory process could be an indicator of degradation of the motor neuron membranes that results in increased Cho MRS signal, as broken down membranes are known to be more “visible” in MRS than structurally intact membranes. As the disease progresses, over a period of time, the motor neuron degeneration in the primary motor cortex and CST causes glial cells to fill up the space left behind by the degenerated membranes leading to an increase in Cho MRS signal. Once both degeneration of membranes in the primary motor cortex and CST and its concomitant glial cell proliferation are completed, no more change in Cho is expected (i.e., flooring of metabolite changes).

No significant group differences were found for Cre in the entire intracranial CST of the ALS group as compared to the control group (Figure 3). Similar findings were reported by previous studies [6], [12], [31], [32] although two studies have reported significantly decreased Cre in ALS [10], [14]. The proton MRS observed Cre signal in the brain at 3T has contributions from creatine and phosphocreatine [7]. The creatine-phosphocreatine and its concomitant ATP-ADP shuttles in the brain cellular energetics ensure a stable concentration of Cre detectable by proton MRS, though it is known to be altered in some brain pathologies [5]. Our observation of no significant difference in Cre indicates that there was no net change in Cre concentration within the cellular components of the entire intracranial CST in ALS. Metabolite ratios to Cre (NAA/Cre and Cho/Cre) were, thus, not included in this analysis.

There were no significant metabolite differences between the entire left and right intracranial CST in both the control and ALS groups. However, the observation of a significant Cho/NAA difference between the left and right centrum semiovale segments only in ALS indicates presence of bilaterally asymmetric alterations along the CST. Furthermore, the above results suggest that the intracranial CST data analysis by segments is a more sensitive evaluation method than by the entire intracranial CST method for detecting regional changes along the CST in ALS. Finding such localized metabolite changes in the early stage of disease will allow us to characterize the disease onset location within the brain and its spread over a period of time in individuals. Also, the observed metabolite bilateral asymmetry in ALS advocates for correlating the metabolite data from the left and right CST separately with clinical measures rather than combining the data from both the sides.

The findings of significant metabolite differences between majority of the intracranial CST segments in each hemisphere in both the control (Table 3; 64 out of 80 comparisons) and ALS groups (Table 4; 41 comparisons) indicate that the metabolite concentrations along the CST segments are significantly heterogeneous. However, the number of significant differences observed in the ALS group was less than that observed in the control group (41 in ALS versus 64 in controls). This could be because the extent of tissue degeneration was different in different segments of the CST in the patient population. These disease-induced varying levels of metabolite alterations along the length of the CST in ALS can be better quantified using the finer segmented CST analysis. However, it should be emphasized that the possible variation in the severity and/or progression of disease in the patient population might also have contributed to the difference in significance levels seen here and further longitudinal studies might be needed to investigate this.

Our results showed significant correlations only between Cho/NAA in the entire intracranial CST of the ALS group and FVC and finger tap rates. At a finer level, significant negative correlations for Cho/NAA between the PCG, CS and PLIC segments in the left CST and clinical measures of FVC and right finger and foot tap rates were observed. The observed negative correlation between Cho/NAA in the left CST and right finger tap rate (r = −0.59, p<0.0001) is an expected association as there is a contralateral anatomical relationship between the regions localizing the upper limb somatotopies in the primary motor cortex and their associated body parts of the upper limbs. However, the similarly expected correlation between Cho/NAA in the right CST and left finger tap rate has a trend towards significance (p = 0.064), possibly due to a slightly less number of patients with left upper limb weakness included in this study (10 patients with left upper limb weakness versus 13 patients with right upper limb weakness). The observed correlations of Cho/NAA in the entire intracranial CST with only finger tap rates (see Results section) and not with the foot tap rates and the syllable repeat (assessing the bulbar muscles) rates can be explained by comparing the CST atlas and the motor homunculus [37] shown in Figure 4. The atlas at the cortical and subcortical levels within the primary cortex only encompasses the voxels localizing the upper limb somatotopies and not the foot and face somatotopic regions. Also inferred from the figure is that the length of the CST atlas between the corona radiata and the cerebral peduncle includes WM fibers fanning out towards the motor cortex regions representing the foot and face somatotopies. However, commingling of WM fibers from the primary motor cortex regions localizing the face, upper limb and lower limb somatotopies along the length of CST between the CS and CP might reduce the sensitivity of detecting metabolite alterations due to a specific dysfunctional body part. It is speculated that the observed correlation between Cho/NAA in the left CST and FVC (see Results section) may be due to this inclusion of WM fibers from the face somatotopic region in the left CST. However, further data analysis from the bilateral motor cortical regions representing the somatotopies of the face and respiratory muscles are needed to better understand such correlations. Further studies are also needed to establish the usefulness of CST segmental analysis in understanding the temporal and spatial (anatomical) patterns of disease progression in ALS. Certainly, our interpretation of the correlations between Cho/NAA and FVC and finger tap rates has to be read with caution as the volume of the processed MRS voxel was about 1 cm^3^ and that was interpolated to 8 mm^3^ volume (2 mm isotropic) for template space analysis.

As mentioned earlier, there is no previous whole intracranial CST MRS study in the literature. However, considering the fact that the CST atlas included data from the primary motor cortex, our results are in agreement with previous findings of significant associations between NAA [12], [16] or NAA/Cre [18], [36], [38], [39] or NAA/Cho [10], [19] or NAA/(Cho+Cre) [11] in the primary motor cortex of patients with ALS and finger tap rate [11], [16] or tests of disease severity [10], [12], [16], [18], [19], [36], [38], [39]. There are, however, a few studies which have reported no correlations between the MRS metrics (measured in this study) in the primary motor cortex and measures of disease severity (ALSFRS-R; [9], [12], [40]) or finger or foot tapping rate [9], [40]. The lack of consistently observed correlations between the MRS metrics and disease severity or UMN function measures in previous MRS studies may be due to inclusion of a heterogeneous patient population with a wide range of disease duration and severity, variable rate of change of metabolites with respect to disease duration and severity, and global measures of disease severity (e.g., ALSFRS-R) not reflecting localized MRS changes.

The MRS results of the CST segments show consistent trends (decreased NAA, and increased Cho, Cre and Cho/NAA in ALS) for the metabolite metrics along the length of the CST bilaterally (Figure 5). The observation of significant changes in Cho/NAA of patients with ALS in all of the CST segments bilaterally indicates that the entire length of the CST between the PMC and CP was affected. In contrast, significant bilaterally asymmetric changes in NAA (only 4 segments in the left CST and no segments in the right CST; Figure 5) and Cho (2 segments in the left and 3 segments in the right; Figure 5) indicate spatial lateralization in the individual metabolite alterations. Furthermore, the above findings suggest presence of two spatially variant metabolic processes in our patient population. The processes include decreased NAA in some segments in the left intracranial CST and increased Cho in the same or other segments, both of which might indicate varying stages of degeneration of neurons and/or axons in the PMC and CST. Although, as stated earlier, there are no methodologically similar previous studies in the literature, our results are qualitatively in agreement with findings from a previous study [19] in that NAA/Cho (Cho/NAA in this study) was consistently lower (higher in this study) across the length of the CST from the precentral gyrus to CP in patients with ALS.

Limitations of this study include that the metabolite and water relaxation times (T1 and T2) were not measured in the MRSI acquisition method used, and therefore, not accounted for in our metabolite signal normalization and quantitation methods. The lengthy relaxation time measurements pose a practical difficulty of including them in our whole-brain MRSI study protocol. Therefore, the reported MRS metrics include possible contributions from altered metabolite relaxation times between the subjects in the patient and control groups. Also, some of the patients participated in this study were treated with riluzole either before participating in this study or at the time of scan. Even though there is no conclusive evidence [41], [42] of altered metabolite concentrations after taking riluzole, the confounding effects of this drug on the metabolite metrics cannot be ruled out completely.

In conclusion, the results of this study demonstrate that significant alterations of proton MRS-observed metabolites occur in the entire intracranial CST, including the primary motor cortex in patients with ALS. The significant correlations between the clinical measures of a body part assessing UMN dysfunction and the MRS metrics evaluated in the relevant body region of the primary motor cortex in the ALS group suggest that the MRS metrics can be potential biomarkers for mapping clinical signs of UMN dysfunction in the relevant body region of the primary motor cortex. The MRS acquisition and processing methods used in this study are currently semi-automated but can be fully automated for use in clinical settings. Finally, this study has shown that the altered ^1^H-MRS metrics in the corticospinal tracts of patients with ALS can be thoroughly characterized by acquiring data from the whole intracranial CST and using an unbiased evaluation method.

## References

[1] Kato S (2008). Amyotrophic lateral sclerosis models and human neuropathology: similarities and differences.. Acta Neuropathol.

[2] Naganska E, Matyja E (2011). Amyotrophic lateral sclerosis - looking for pathogenesis and effective therapy.. Folia Neuropathol.

[3] Filippi M, Agosta F, Abrahams S, Fazekas F, Grosskreutz J (2010). EFNS guidelines on the use of neuroimaging in the management of motor neuron diseases.. Eur J Neurol.

[4] Turner MR, Modo M (2010). Advances in the application of MRI to amyotrophic lateral sclerosis.. Expert Opin Med Diagn.

[5] Govindaraju V, Young K, Maudsley AA (2000). Proton NMR chemical shifts and coupling constants for brain metabolites.. NMR Biomed.

[6] Bowen BC, Pattany PM, Bradley WG, Murdoch JB, Rotta F (2000). MR imaging and localized proton spectroscopy of the precentral gyrus in amyotrophic lateral sclerosis.. AJNR Am J Neuroradiol.

[7] Chan S, Shungu DC, Douglas-Akinwande A, Lange DJ, Rowland LP (1999). Motor neuron diseases: comparison of single-voxel proton MR spectroscopy of the motor cortex with MR imaging of the brain.. Radiology.

[8] Charil A, Corbo M, Filippi M, Kesavadas C, Agosta F (2009). Structural and metabolic changes in the brain of patients with upper motor neuron disorders: a multiparametric MRI study.. Amyotroph Lateral Scler.

[9] Kalra S, Hanstock CC, Martin WR, Allen PS, Johnston WS (2006). Detection of cerebral degeneration in amyotrophic lateral sclerosis using high-field magnetic resonance spectroscopy.. Arch Neurol.

[10] Pohl C, Block W, Karitzky J, Traber F, Schmidt S (2001). Proton magnetic resonance spectroscopy of the motor cortex in 70 patients with amyotrophic lateral sclerosis.. Arch Neurol.

[11] Rooney WD, Miller RG, Gelinas D, Schuff N, Maudsley AA (1998). Decreased N-acetylaspartate in motor cortex and corticospinal tract in ALS.. Neurology.

[12] Sarchielli P, Pelliccioli GP, Tarducci R, Chiarini P, Presciutti O (2001). Magnetic resonance imaging and 1H-magnetic resonance spectroscopy in amyotrophic lateral sclerosis.. Neuroradiology.

[13] Unrath A, Ludolph AC, Kassubek J (2007). Brain metabolites in definite amyotrophic lateral sclerosis. A longitudinal proton magnetic resonance spectroscopy study.. J Neurol.

[14] Block W, Traber F, Flacke S, Jessen F, Pohl C (2002). In-vivo proton MR-spectroscopy of the human brain: assessment of N-acetylaspartate (NAA) reduction as a marker for neurodegeneration.. Amino Acids.

[15] Rule RR, Suhy J, Schuff N, Gelinas DF, Miller RG (2004). Reduced NAA in motor and non-motor brain regions in amyotrophic lateral sclerosis: a cross-sectional and longitudinal study.. Amyotroph Lateral Scler Other Motor Neuron Disord.

[16] Mitsumoto H, Ulug AM, Pullman SL, Gooch CL, Chan S (2007). Quantitative objective markers for upper and lower motor neuron dysfunction in ALS.. Neurology.

[17] Yin H, Lim CC, Ma L, Gao Y, Cai Y (2004). Combined MR spectroscopic imaging and diffusion tensor MRI visualizes corticospinal tract degeneration in amyotrophic lateral sclerosis.. J Neurol.

[18] Wang S, Poptani H, Woo JH, Desiderio LM, Elman LB (2006). Amyotrophic lateral sclerosis: diffusion-tensor and chemical shift MR imaging at 3.0 T.. Radiology.

[19] Pyra T, Hui B, Hanstock C, Concha L, Wong JC (2010). Combined structural and neurochemical evaluation of the corticospinal tract in amyotrophic lateral sclerosis.. Amyotroph Lateral Scler.

[20] Brooks BR, Miller RG, Swash M, Munsat TL (2000). El Escorial revisited: revised criteria for the diagnosis of amyotrophic lateral sclerosis.. Amyotroph Lateral Scler Other Motor Neuron Disord.

[21] Cedarbaum JM, Stambler N, Malta E, Fuller C, Hilt D (1999). The ALSFRS-R: a revised ALS functional rating scale that incorporates assessments of respiratory function. BDNF ALS Study Group (Phase III).. J Neurol Sci.

[22] Kent-Braun JA, Walker CH, Weiner MW, Miller RG (1998). Functional significance of upper and lower motor neuron impairment in amyotrophic lateral sclerosis.. Muscle Nerve.

[23] Sharma KR, Saigal G, Maudsley AA, Govind V (2011). ^1^H MRS of basal ganglia and thalamus in amyotrophic lateral sclerosis.. NMR Biomed.

[24] Govind V, Gold S, Kaliannan K, Saigal G, Falcone S (2010). Whole-brain proton MR spectroscopic imaging of mild-to-moderate traumatic brain injury and correlation with neuropsychological deficits.. J Neurotrauma.

[25] Maudsley AA, Darkazanli A, Alger JR, Hall LO, Schuff N (2006). Comprehensive processing, display and analysis for in vivo MR spectroscopic imaging.. NMR Biomed.

[26] Collins DL, Zijdenbos AP, Kollokian V, Sled JG, Kabani NJ (1998). Design and construction of a realistic digital brain phantom.. IEEE Trans Med Imaging.

[27] Smith SA, Levante TO, Meier BH, Ernst RR (1994). Computer simulations in magnetic resonnace. An object oriented programming approach.. Journal of Magnetic Resonance Ser A.

[28] Oishi K, Faria A, Jiang H, Li X, Akhter K (2009). Atlas-based whole brain white matter analysis using large deformation diffeomorphic metric mapping: application to normal elderly and Alzheimer's disease participants.. Neuroimage.

[29] Zhang Y, Zhang J, Oishi K, Faria AV, Jiang H (2010). Atlas-guided tract reconstruction for automated and comprehensive examination of the white matter anatomy.. Neuroimage.

[30] Kaufmann P, Pullman SL, Shungu DC, Chan S, Hays AP (2004). Objective tests for upper motor neuron involvement in amyotrophic lateral sclerosis (ALS).. Neurology.

[31] Gredal O, Rosenbaum S, Topp S, Karlsborg M, Strange P (1997). Quantification of brain metabolites in amyotrophic lateral sclerosis by localized proton magnetic resonance spectroscopy.. Neurology.

[32] Schuff N, Rooney WD, Miller R, Gelinas DF, Amend DL (2001). Reanalysis of multislice (1)H MRSI in amyotrophic lateral sclerosis.. Magn Reson Med.

[33] Block W, Karitzky J, Traber F, Pohl C, Keller E (1998). Proton magnetic resonance spectroscopy of the primary motor cortex in patients with motor neuron disease: subgroup analysis and follow-up measurements.. Arch Neurol.

[34] Maudsley AA, Domenig C, Sheriff S (2010). Reproducibility of serial whole-brain MR spectroscopic imaging.. NMR Biomed.

[35] Simmons ML, Frondoza CG, Coyle JT (1991). Immunocytochemical localization of N-acetyl-aspartate with monoclonal antibodies.. Neuroscience.

[36] Ellis CM, Simmons A, Andrews C, Dawson JM, Williams SC (1998). A proton magnetic resonance spectroscopic study in ALS: correlation with clinical findings.. Neurology.

[37] Penfield W, Boldrey E (1937). Somatic motor and sensory representation in the cerebral cortex of man as studied by electrical stimulation.. Brain.

[38] Abe K, Takanashi M, Watanabe Y, Tanaka H, Fujita N (2001). Decrease in N-acetylaspartate/creatine ratio in the motor area and the frontal lobe in amyotrophic lateral sclerosis.. Neuroradiology.

[39] Sivak S, Bittsansky M, Kurca E, Turcanova-Koprusakova M, Grofik M (2010). Proton magnetic resonance spectroscopy in patients with early stages of amyotrophic lateral sclerosis.. Neuroradiology.

[40] Kalra S, Tai P, Genge A, Arnold DL (2006). Rapid improvement in cortical neuronal integrity in amyotrophic lateral sclerosis detected by proton magnetic resonance spectroscopic imaging.. J Neurol.

[41] Kalra S, Cashman NR, Genge A, Arnold DL (1998). Recovery of N-acetylaspartate in corticomotor neurons of patients with ALS after riluzole therapy.. Neuroreport.

[42] Bradley WG, Bowen BC, Pattany PM, Rotta F (1999). 1H-magnetic resonance spectroscopy in amyotrophic lateral sclerosis.. J Neurol Sci.

